# Teaching ophthalmic microsurgical skills: conventional microscopy versus heads-up visualization

**DOI:** 10.1186/s12909-026-10269-9

**Published:** 2026-08-31

**Authors:** Franziska Eckardt, Maximilian -Joachim Gerhardt, Siegfried Priglinger

**Affiliations:** Department of Ophthalmology, LMU University Hospital, LMU Munich, Munich, Germany

**Keywords:** Retina, Cataract, Ophthalmology, Three-dimensional surgery

## Abstract

**Background:**

To compare training effects between conventional operating microscope and a3D heads-up system using the EyeSi^®^ simulator (Haag-Streit, Köniz, Switzerland) in medical students and residents without prior microsurgical experience.

**Methods:**

In a prospective single-center crossover study, 16 medical students with ophthalmological exposure and 16 residents completed standardized cataract and vitrectomy modules on the EyeSi^®^ simulator using both a conventional ocular microscope and a 3D display. Objective performance parameters and subjective evaluations (questionnaire) regarding safety, handling, comfort, learning progress, as well as interaction were assessed.

**Results:**

In the vitrectomy simulation, no significant difference in scores was found between visualization systems (*p* = 0.126), whereas in the cataract simulation, the conventional microscope achieved significantly higher scores (*p* = 0.023). The overall task scores did not differ significantly (*p* = 0.376). Subjectively, the 3D system was rated superior regarding control (*p* = 0.013), instrument handling (*p* = 0.033), movement precision (*p* < 0.001), comfort, and instructor interaction (*p* < 0.001). Students tended to prefer the 3D system, whereas residents more often preferred the conventional microscope, although this difference was not statistically significant (*p* = 0.183). All participants reported that skills acquired with the 3D system could be perceived transferability to the conventional microscope.

**Conclusion:**

The 3D display achieved comparable simulator performance in the vitrectomy module but lower performance in the cataract module compared with the conventional microscope, while offering perceived didactic and ergonomic advantages. The differing preferences between students and residents may suggest a possible habituation effect due to prior slit-lamp use and familiarity with conventional visualization techniques. As digital visualization systems are becoming increasingly integrated into contemporary ophthalmic surgery, early exposure to heads-up technology during simulator-based training may help familiarize trainees with modern visualization techniques. Future clinical studies are required to determine whether these simulator-based findings translate into improved operative performance.

**Supplementary Information:**

The online version contains supplementary material available at https://doi.org/10.1186/s12909-026-10269-9.

## Introduction

Simulation-based surgical training has become an integral component of ophthalmic education, particularly with the increasing adoption of dry-lab methodologies that allow skill acquisition without patient risk [1–3]. Dry-lab models, including synthetic eyes, three-dimensional printed structures, and virtual reality (VR) simulators, enable trainees to practice instrument handling, spatial orientation, and specific surgical steps such as capsulorhexis, vitrectomy, suturing techniques, or endolaser photocoagulation in a controlled environment [3–8]. The relevance of these training modalities has further increased following the reduction of clinical training opportunities during the COVID-19 pandemic [1, 2, 9]. Previous studies and systematic reviews have demonstrated that simulation-based training improves technical performance and is especially beneficial during early stages of surgical education [4, 10, 11].

Traditionally, ophthalmic microsurgery is performed using a binocular microscope with oculars. In recent years, however, three-dimensional (3D) heads-up display systems have been increasingly introduced into clinical practice [12–16]. These systems project a stereoscopic surgical image onto a high-resolution monitor, allowing surgeons to operate in a more ergonomic posture while potentially enhancing depth perception, visualization, and teaching conditions. Prospective studies in vitreoretinal surgery have reported high satisfaction rates among both trainees and instructors and suggest potential educational advantages of 3D visualization systems compared with conventional microscopy [12–14, 16–19].

Despite these promising findings, most existing studies have focused on surgeons with substantial experience in slit-lamp examination and operating microscopes [13, 20–25]. Consequently, it remains unclear whether the advantages attributed to 3D heads-up systems also extend to novices without prior microsurgical experience. Clarifying this question is particularly relevant for the design of early ophthalmic training curricula.

One of the most widely used VR simulators in ophthalmology is the EyeSi^®^ Surgical Simulator (Haag-Streit, Köniz, Switzerland). The EyeSi platform provides validated training modules for cataract and vitreoretinal surgery, objective performance metrics, and the possibility of repeated practice. Its construct validity and educational benefit have been well established, and several studies have demonstrated a perceived transferability of simulator-acquired skills to the operating room, particularly among trainees with limited surgical experience [5, 7, 8, 10, 26–29].

The aim of the present study was to compare the training effects of a conventional ocular microscope and a 3D heads-up display system using the EyeSi^®^ surgical simulator in medical students and early-career ophthalmology residents without prior microsurgical experience. Objective performance parameters and subjective perceptions of safety, comfort, handling, as well as learning progress were assessed to determine which visualization system may be more suitable for the introduction to microsurgical training.

## Material & methods

### Study design

This study was designed as a single-center, cross-sectional, intraindividual comparative study conducted at the Department of Ophthalmology, Ludwig Maximilian University (LMU) Munich. This study was approved by the Ethics Committee of the Medical Faculty of LMU Munich (study ID: 25–0815 ) and adhered to the principles of the Declaration of Helsinki.

### Participants

Two groups, medical students during ophthalmology rotation and ophthalmology residents in their early years of training were recruited. Inclusion criteria were the absence of prior microsurgical experience and no previous structured training with ophthalmic surgical simulators. Medical students were recruited during or after completion of the ophthalmology curriculum to ensure basic anatomical and ophthalmological knowledge.

### Simulator modules

**Fig. 1:**
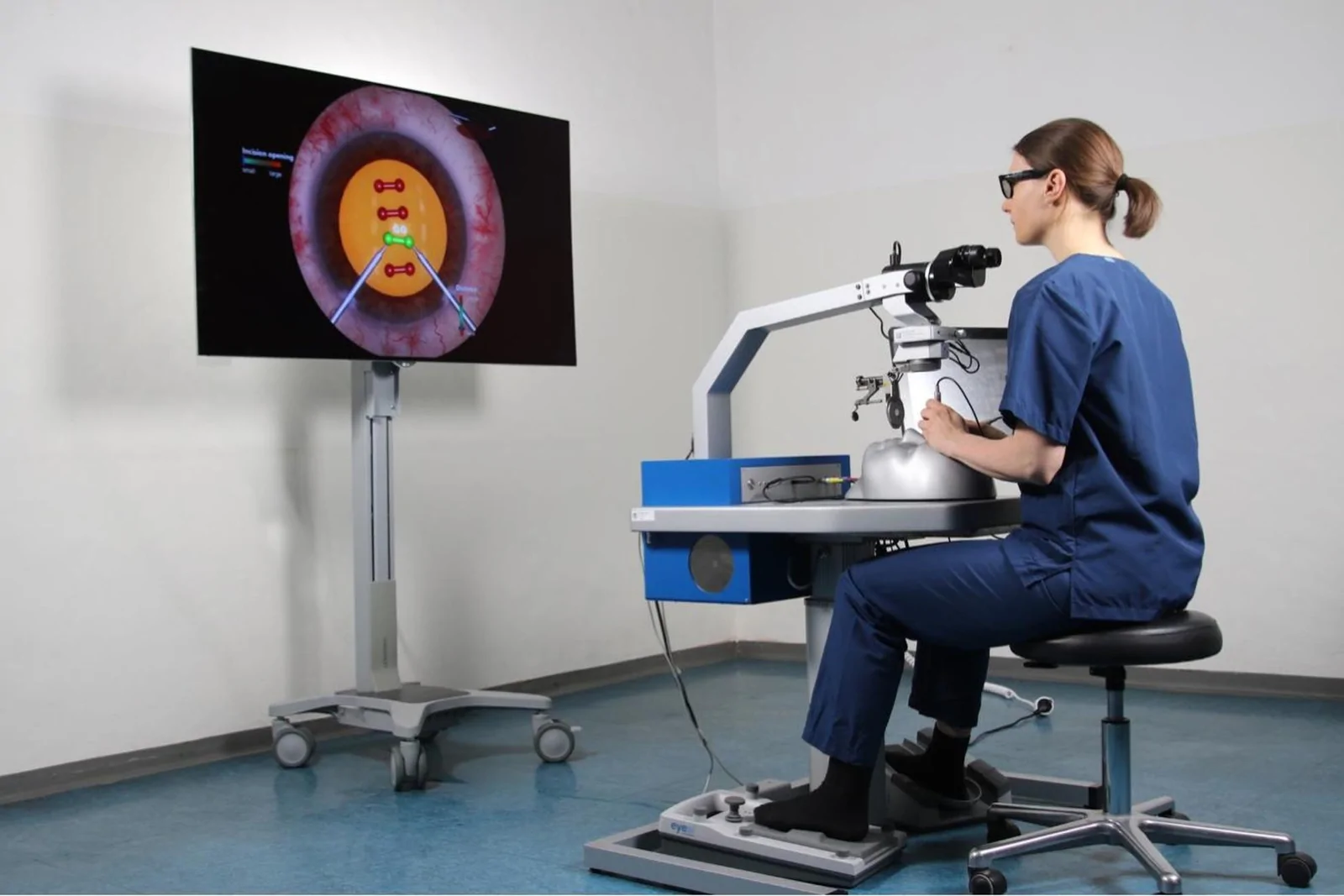
Experimental setup of the EyeSi^®^ Surgical Simulator (Haag-Streit, Köniz, Switzerland) with the 3D heads-up visualization system used for microsurgical simulation training

All participants performed standardized training tasks on the EyeSi^®^ Surgical Simulator (Haag-Streit, Köniz, Switzerland, Fig. 1). Training modules were selected to represent basic, beginner-appropriate skills and isolated steps of ophthalmic surgery requiring comparable levels of difficulty. The tasks were designed to assess fundamental instrument handling, precision, and visuomotor coordination.

Each participant completed training sessions using both visualization systems available on the simulator:

1. a conventional microscopic visualization with oculars, and.
2. a three-dimensional heads-up display system.

### Study procedure

**Fig. 2:**
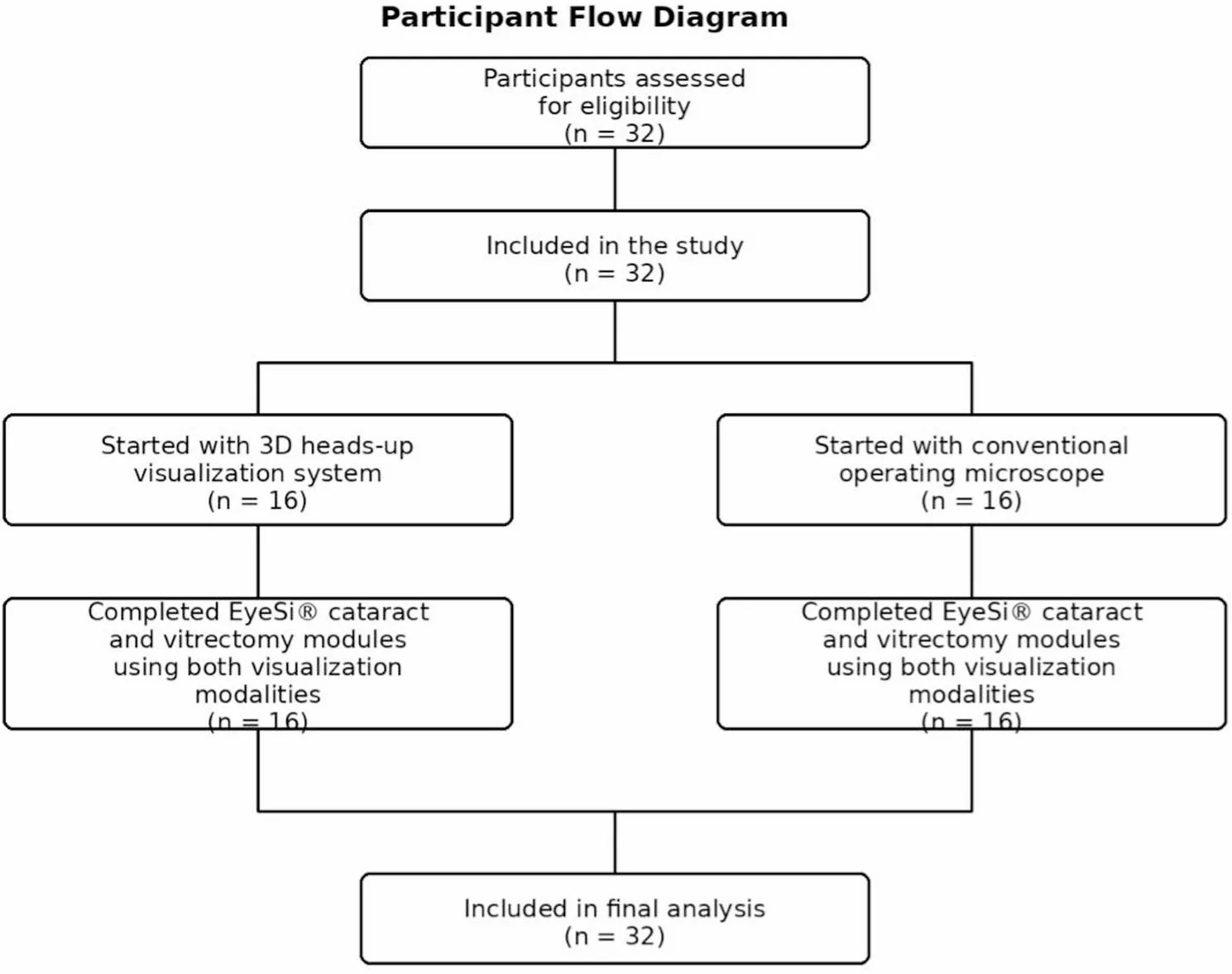
Participant flow diagram

Participants were enrolled consecutively as they became available. To minimize potential order effects while maintaining balanced intervention sequences throughout the recruitment period, participants were assigned to the starting visualization system using an alternating allocation scheme. This approach ensured that both intervention sequences remained equally represented at all stages of recruitment, even if recruitment had ended earlier than anticipated. Consequently, 16 participants started with the 3D heads-up visualization system and 16 with the conventional microscope (Fig. 2). As the allocation sequence was predetermined, allocation concealment was not possible. Blinding of participants and investigators was not feasible because of the obvious differences between the two visualization systems. Participants were informed of the starting visualization system immediately before commencing the simulator session. Objective performance outcomes were recorded automatically by the EyeSi^®^ surgical simulator. Each participant completed a predefined set of training tasks with each system during a single study session. The total duration of participation was approximately one hour.

Objective refraction was performed in all participants before study participation. Participants with high anisometropia (> 2.5 diopters ) were not present in the study cohort.

For each task, the EyeSi^®^ simulator automatically generated objective performance metrics, including overall performance scores, precision parameters, error rates, and task completion times. Prior to the formal training tasks, all participants completed a standardized 10-minute familiarization phase, comprising 5 min of practice with the conventional operating microscope and 5 min with the 3D heads-up display. During this period, participants received a brief explanation of the EyeSi^®^ surgical simulator and were allowed to acclimatize to the system. This included hands-on practice with the microscope foot pedal, instrument handling, use of the three-dimensional monitor, and adjustment of the conventional microscopic visualization. Familiarization was conducted using a basic introductory exercise that was excluded from the subsequent performance analysis.

**Fig. 3:**
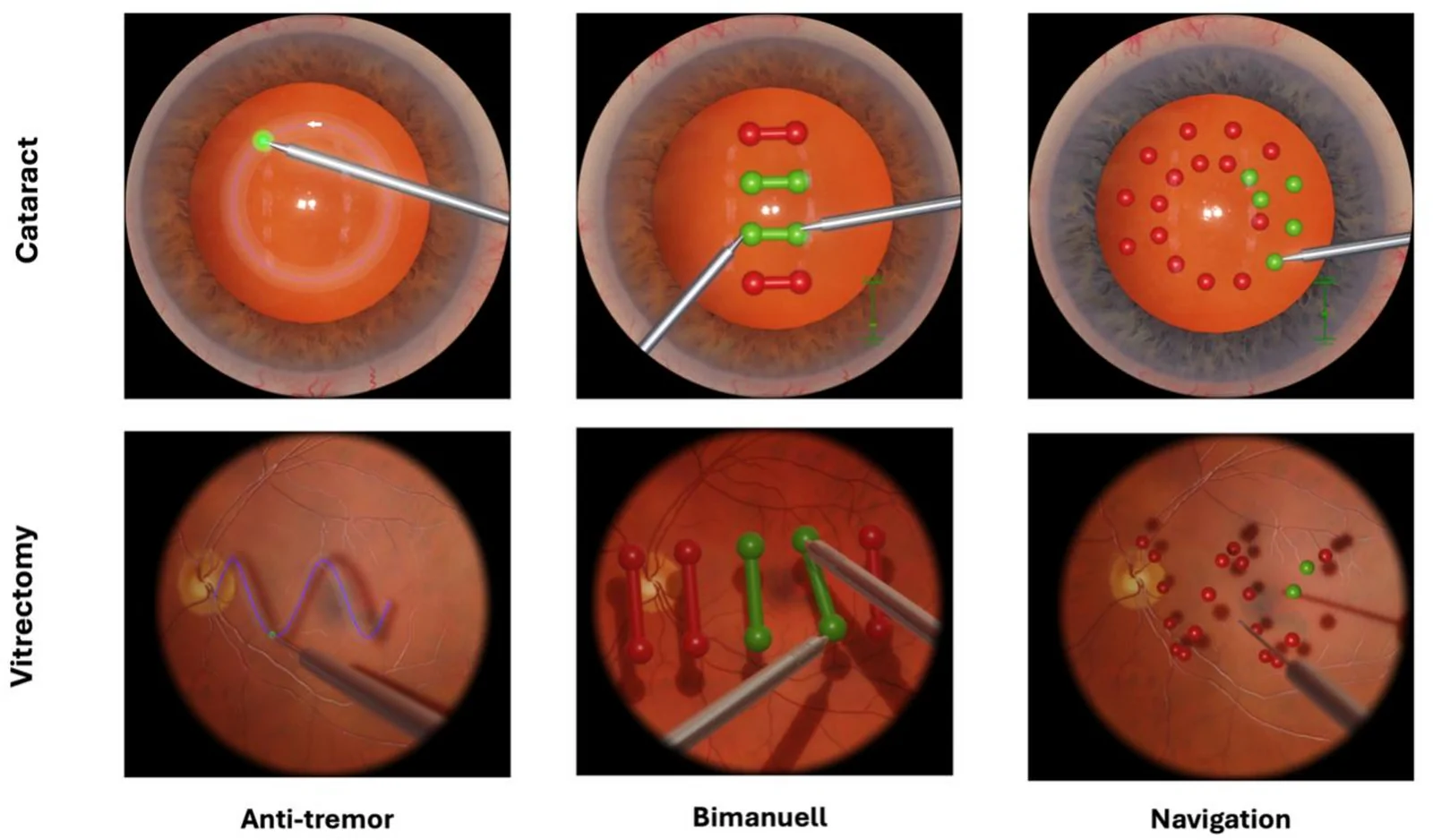
EyeSi^®^ Surgical Simulator tasks performed by the participants during standardized cataract and vitreoretinal training modules

After completion of the familiarization phase, each participant performed three training exercises in a cataract surgery setting and three in a vitreoretinal surgery setting (Fig. 3). The exercises were deliberately selected to assess comparable fundamental microsurgical skills across both settings. Specifically, tasks focused on anti-tremor control, bimanual coordination, and spatial navigation. Between the two visualization modalities, participants completed a standardized 5-minute break during which they were instructed to focus on distant objects to reduce visual and accommodative fatigue before continuing with the second visualization system.

To control for potential learning and order effects, a balanced crossover design was applied. Participants who started with the heads-up display in the cataract module and subsequently performed the same exercises using the conventional microscopic visualization completed the vitreoretinal module in the reverse order, first using the ocular microscope followed by the 3D heads-up display system. This approach ensured that each participant used both visualization systems in both surgical settings while minimizing systematic sequence bias. All participants were supervised by the same instructor (FE).

### Subjective assessment

After completing the training sessions, participants completed a study-specific standardized questionnaire (Supplementary material 1; original questionnaire translated into English). Because no validated questionnaire addressing the comparison of conventional ocular microscopy and 3D heads-up visualization in simulator-based microsurgical training was available, a study-specific questionnaire was developed. Prior to study initiation, the questionnaire was reviewed by several experienced ophthalmic surgeons routinely using both conventional microscopes and 3D heads-up visualization systems to assess its clarity, relevance, and content. No formal pilot testing or psychometric validation was performed. The questionnaire assessed subjective perceptions of safety and understanding, comfort and ergonomics, handling, and perceived learning progress for each visualization system. Responses were recorded using a five-point Likert scale ranging from 1 (“strongly disagree”) to 5 (“strongly agree”). Additional comparative items asked participants to indicate which system they perceived as easier to use, safer, more comfortable, and more suitable for beginners. Open-ended questions provided the opportunity for qualitative feedback and suggestions for improvement.

### Statistical analysis

Data analysis was performed using paired statistical methods to account for the intraindividual crossover design. Normality of each outcome variable was assessed using the Shapiro–Wilk test. Depending on the distribution of the respective variable, paired t-tests or Wilcoxon signed-rank tests were used to compare objective performance metrics and subjective outcomes between the two visualization systems. Individual questionnaire items based on the 5-point Likert scale were analyzed using the Wilcoxon signed-rank test. For descriptive purposes, an overall questionnaire score was calculated as an exploratory summary across all questionnaire items. Descriptive statistics were calculated for all variables, and statistical significance was defined as α = 0.05. Recruitment was limited by the temporary availability of the 3D heads-up visualization system, which was provided on loan for a restricted period. Therefore, no formal a priori sample size calculation was performed. To characterize the statistical properties of the available sample, a sensitivity analysis was conducted using G*Power (version 3.1) for a two-sided paired-samples t-test. Assuming an alpha level of 0.05 and a statistical power of 80%, the available sample size of 32 participants allowed detection of a standardized within-participant effect size of approximately Cohen’s dz = 0.51, corresponding to a medium effect. As no comparable studies were available, no empirical effect-size estimate could be used for planning purposes.

## Results

### Study population

A total of 32 participants were included in the study, comprising 16 medical students (50%) and 16 early-career ophthalmology residents (50%) without prior microsurgical experience (Table 1). All participants completed the full study protocol, including the familiarization phase and all assigned training exercises using both visualization systems. No technical difficulties or adverse events occurred during the training sessions.

**Table 1:** Participant characteristics

Characteristic	Medical students (*n* = 16)	Residents (*n* = 16)	Overall ( *n* = 32)
Age (year)	26.09 ± 1.04	28.56 ± 1.86	27.56 ± 1.99
Gender (male/female)	8/8	8/8	16/16
Objective refraction: sphere	OD: − 1.43 ± 2.46 OS: − 1.45 ± 2.59	OD:- 0.88 ± 2.47 OS: -1.04 ± 1.94	OD: -1.79 ± 2.34 OS: -1.26 ± 2.28
Objective refraction: cylinder	OD: -0.53 ± 0.39 OS: -0.48 ± 0.73	OD: -0.52 ± 0.31 OS: -0.65 ± 0.28	OD: -0.53 ± 0.35 OS: -0.56 ± 0.56
Previous ophthalmic exposure	16/16	16/16	32/32
Year of residency	0	1.19 ± 1.42	1.19 ± 1.42
Previous microsurgical experience (real world OR)	0/16	0/16	0/32
Previous wetlab experience cataract	2/16	8/16	10/32
Previous wetlab experience retina	0/16	0/16	0/32
Routine slit-lamp use > 1 year	0/16	16/16	16/32
EyeSi experience	0	0	0

### Objective simulator-based performance

Objective performance metrics generated by the EyeSi^®^ surgical simulator were available for all exercises and visualization systems. The achieved scores in the respective modules are presented in Table 2. The overall performance scores did not differ between the heads-up display and the conventional microscopic visualization (*p* = 0.376). Focusing on the cataract module, the subjects achieved significantly higher scores using the conventional microscopic than with the 3D-heads-up display (*p* = 0.023). Across the vitrectomy module there was no significant difference between conventional microscopic visualization and the 3D- heads-up display (*p* = 0.126). If the individual tasks within the cataracts and vitrectomy modules are considered separately, a significant difference was observed only in the navigation task, both in the cataract and in the vitrectomy module (*p* = 0.023, *p* = 0.025). No significant sequence effect was observed. Participants who started with the 3D heads-up system achieved overall simulator scores comparable to those who started with the conventional microscope (*p* = 0.608, Cohen’s d = 0.18, 95% CI − 0.51 to 0.88).

**Table 2:** Mean simulation scores

Task	Mean Score conventional	Mean Score 3D-system	Cohen’s d 95% CI	*P*- value
Cataract Simulation				
1. Bimanual	48.69 ± 30.85	46.06 ± 21.95	-0.9 [-0.44;0.26]	0.616
2. Navigation	65.47 ± 19.71	51.00 ± 29.40	− 0.43 [-0.78; -0.06]	**0.022**
3. Anti-Tremor	33.63 ± 29.51	22.62 ± 23.27	-0.33 [-0.69; 0.03]	0.070
4. Total score	147.16 ± 56.05	119.69 ± 57.32	**-**0.42 [-0.78;0.06]	**0.023**
Vitrectomy Simulation				
1. Bimanual	68.38 ± 23.84	69.97 ± 26.14	0.05 [-0.29;0.4].	0.765
2. Navigation	56.16 ± 11.34	51.38 ± 11.89	-0.24 [-0.77;-0.05]	**0.025**
3. Anti-Tremor	15.09 ± 21–42	9.72 ± 14.33	-0.27 [-0.62;0.09]	0.142
4. Total score	141.81 ± 43.04	131.06 ± 38.48	0.28 [-0.08;0.63]	0.126

Standardized effect sizes (Cohen's d) with corresponding 95% confidence intervals for paired comparisons between the 3D heads-up system and the conventional microscope. Positive values indicate higher scores using the 3D heads-up system, whereas negative values indicate higher scores using the conventional microscope

Bold: significant *p*-values

### Subjective assessment

Subjective evaluations were completed by all participants using the standardized questionnaire. Participants reported no significant difference in overall questionnaire scores between the two visualization systems. The overall score for the 3D visualization system was 70 ± 7.11 and the overall score for the conventional microscope was 68.31 ± 9.53 (*p* = 0.376). Analysis of the individual questionnaire items that were assessed identically for both systems revealed no significant difference in the overall evaluation. However, significant differences were found for items 1, 11, and 12, with the 3D-visualization system achieving higher ratings in these domains:

- Question 1: I felt in control at all times during the task (*p* = 0.013).
- Question 11: I found the handling of the instruments easy (*p* = 0.033).
- Question 12: I was able to perform my movements with precision (*p* < 0.001).

Exploratory subgroup analysis revealed no significant difference in cataract performance between visualization systems among medical students (*p* = 0.718), whereas ophthalmology residents achieved significantly higher cataract scores with the conventional microscope (*p* = 0.003).

### Comparative preference

When directly comparing both visualization systems, participants indicated a significant preference for the 3D visualization system over the conventional microscopic visualization in terms of teacher-trainee interaction (*p* < 0.001) and comfort (*p* < 0.001). In the comparative assessment of task difficulty, perceived learning progress, and sense of safety, participants did not demonstrate a statistically significant preference for either visualization system (*p* = 0.572, *p* = 1.0, *p* = 0.087). Regarding the question of which system participants would recommend for entry into microsurgery, no significant difference was observed in the overall analysis; however, when stratified by position, a greater proportion of students tended to prefer the 3D system (*p* = 0.267), whereas more residents recommended the conventional microscopic visualization (*p* = 0.388), although this difference did not reach statistical significance (*p* = 0.18).

All participants indicated that the skills acquired using the 3D visualization method could subsequently be applied when using the conventional microscopic visualization.

### Qualitative feedback

Open-ended responses provided additional qualitative insights into the advantages and limitations of each visualization system. Participants highlighted specific aspects related to ergonomics, depth perception, and ease of orientation. Several participants reported that operating with the conventional microscopic visualization felt more familiar and therefore perceived as safer, as it more closely resembles working at the slit lamp; this perception was expressed primarily by ophthalmology residents. Questionnaire feedback indicated that a subset of participants initially perceived the heads-up display as unfamiliar, possibly owing to its differences from the conventional visual experience and handling compared with the conventional slit-lamp use.

Interestingly, participants differed considerably in their perception of which system provided better stereoscopic vision, and no consistent preference for either system emerged. One participant specifically reported preferring the conventional microscopic visualization for the cataract module and the 3D system for the vitrectomy module, a distinction that was also consistent with the respective performance scores achieved.

All participants supported the integration of the simulator into the surgical training curriculum for ophthalmology residents.

## Discussion

Previous studies investigating heads-up display systems in ophthalmic surgery have predominantly focused on experienced surgeons and trainees with substantial prior exposure to conventional microscopy [13, 20, 23–25]. In these populations, heads-up systems have been associated with improved ergonomics, high user satisfaction, and comparable or superior surgical performance [14, 17, 21, 30]. However, evidence regarding their role during the earliest stages of surgical training remains limited.

The present study extends existing knowledge by specifically examining novice learners without prior microsurgical experience. To the best of our knowledge, this is the first study using the 3D heads-up system of the new EyeSi^®^ simulator. Although overall simulator performance scores did not differ significantly between visualization modalities, a more differentiated analysis provides important educational insights. Notably, although the cataract module showed higher scores with conventional microscopic visualization, this effect is likely attributable to familiarity bias. Many participants - particularly ophthalmology residents - were accustomed to slit-lamp examination, which closely resembles conventional microscopic visualization. An exploratory subgroup analysis showed that medical students performed similarly with both visualization systems, whereas residents achieved significantly higher cataract scores using the conventional microscope. Although this finding is consistent with a possible habituation effect resulting from prior slit-lamp experience, this interpretation remains exploratory and should be confirmed in larger studies. Qualitative feedback strongly supported this interpretation, as the conventional microscope was frequently described as more familiar and therefore perceived as safer. These findings suggest that early performance differences may primarily reflect pre-existing visuomotor conditioning rather than an intrinsic superiority of the visualization system itself.

Importantly, the absence of significant differences in overall performance suggest that novice participants were able to perform comparably using both visualization modalities within the EyeSi^®^. From an educational perspective, these comparable simulator outcomes, combined with additional ergonomic and didactic advantages, are highly relevant.

Subjective assessments further revealed distinct strengths of the 3D heads-up system. Participants rated the 3D visualization significantly higher in terms of teacher–trainee interaction and comfort.

The shared visualization provided by a large stereoscopic monitor likely enhances instructional clarity, facilitates real-time feedback, and improves communication between instructor and trainee. This pedagogical advantage represents a central finding of the study.

Crucially, all participants reported that skills and guidance acquired during heads-up training could be directly perceived transferability to the conventional ocular microscope within the simulator environment. These subjective perceptions suggest that participants did not experience relevant barriers when switching between visualization systems during simulator training. However, the present study evaluated simulator performance only. Whether these perceived benefits translate into improved technical performance or learning outcomes in the operating room remains unknown and should be investigated in future clinical studies.

When combined with the significantly improved perception of teacher–trainee interaction, this finding underscores the potential of heads-up systems as powerful tools in microsurgical education, particularly during the early stages of training. Interestingly, students showed a stronger tendency to recommend the 3D system for entry into microsurgery, whereas residents more frequently favored the conventional microscope, although this difference was not statistically significant. This divergence likely reflects the influence of prior exposure and professional habituation rather than objective performance differences. It may also suggest that younger learners without established visuomotor routines adapt more readily to novel visualization technologies. The observed trend may reflect differences in previous exposure and professional habituation; however, this explanation remains speculative and requires confirmation in larger studies.

Beyond the educational setting, existing literature demonstrates that 3D visualization systems offer enhanced intraoperative visualization in selected procedures and enable advanced digital integration, including overlay imaging and improved visualization of intraoperative data [17, 31, 32]. As ophthalmic surgery continues to evolve toward digitally assisted and image-guided techniques, 3D microscopy is likely to play an increasingly important role in future surgical practice [15, 17]. However, in countries with long residency training programs, substantial habituation to slit-lamp examination and conventional microscopy may contribute to an initial learning curve when transitioning to 3D systems. The present findings – particularly the greater acceptance and ease of use reported by students –.

raise the possibility that early exposure to 3D visualization through simulator-based training could facilitate adaptation to digital visualization systems. Integrating heads-up systems into early microsurgical education could therefore facilitate a smoother transition to modern digital operating environments later in clinical practice. In addition, the ergonomic benefits of an upright posture and reduced neck flexion are well documented in ophthalmic surgery, and early exposure to ergonomically favorable systems may promote healthier long-term working habits [12, 15, 17, 21] .

Several limitations should be acknowledged. The single-center design and moderate sample size may limit the generalizability of the findings. The sample size was constrained by the limited availability of the loaned 3D visualization system. Consequently, although the study was sufficiently informative to detect medium within-participant effects, it may have been underpowered to detect smaller differences. Likewise, subgroup analyses comparing medical students and residents should be regarded as exploratory and interpreted with caution.

Furthermore, the present study assessed performance exclusively within the EyeSi^®^ simulator. Although simulator training is an established component of ophthalmic surgical education, simulator performance cannot be assumed to directly reflect operative performance or clinical competency. Therefore, whether the observed findings translate into improved surgical performance in the operating room remains to be determined in future longitudinal clinical studies. Additionally, the subjective questionnaire used in this study was developed specifically for the present investigation and was not formally validated.

A formal stereopsis assessment was not performed. Although none of the participants had a high anisometropia on objective refraction, subtle differences in binocular vision cannot be excluded and may have influenced performance with the 3D visualization system. The relatively short duration of each simulator session may have limited participants’ ability to fully experience physical discomfort or ergonomic strain. Consequently, subjective findings should be interpreted as exploratory and considered complementary to the objective simulator performance measures. Longitudinal studies are needed to determine whether early exposure to 3D visualization influences learning curves, skill retention, or operating room performance. Nevertheless, the present findings indicate that the 3D heads-up visualization system was associated with perceived ergonomic and pedagogical advantages while yielding broadly comparable objective simulator performance in novice learners.

Given the growing integration of digital visualization systems into ophthalmic surgery, incorporating heads-up technology into early simulator-based training may not only reflect contemporary surgical practice but also enhance the educational environment.

In conclusion, while both visualization modalities allow effective acquisition of fundamental microsurgical skills, the 3D heads-up display provides meaningful advantages in ergonomics and instructional interaction. These findings suggest that the 3D heads-up system represents a promising adjunct to simulator-based microsurgical training. Given the increasing adoption of digital visualization systems in ophthalmic surgery, familiarizing trainees with heads-up technology early in their learning curve may help prepare them for modern surgical practice. Future clinical studies are warranted to determine whether the subjective advantages observed during simulation translate into measurable improvements in surgical training and operative performance.

## Supplementary Information


Supplementary Material 1.


## Data Availability

The datasets used and analysed during the current study available from the corresponding author on reasonable request.

## Abbreviations

3D: Three-dimensional
VR: Virtual reality

## References

[1] Chhabra K, Khanna V, Vedachalam R, Sindal M. RetiSurge - Enabling Dry Lab vitreoretinal surgical training during COVID-19 pandemic. Indian J Ophthalmol. 2021;69:982–4. 10.4103/IJO.IJO_2729_20. 33727472 PMC8012966

[2] Kaushik J, Chaitanya Y, Kumar A, et al. Prevalence and effectiveness of innovative techniques in ophthalmic surgical training during COVID-19 pandemic in India. Indian J Ophthalmol. 2021;69:3704. 10.4103/IJO.IJO_1886_21. 34827027 PMC8837362

[3] Yang L, Al-Ani A, Bondok MS, et al. The impact of extended reality simulators on ophthalmic surgical training and performance: a systematic review and meta-analysis of 17,623 eyes. Eye (Lond). 2025;39:1700–9. 10.1038/S41433-025-03722-4. 40021780 PMC12130267

[4] Lee R, Raison N, Lau WY, et al. A systematic review of simulation-based training tools for technical and non-technical skills in ophthalmology. Eye (Lond). 2020;34:1737–59. 10.1038/S41433-020-0832-1. 32203241 PMC7609318

[5] Dormegny L, Yaïci R, Koestel E, et al. Global trends and practice patterns in virtual reality simulation training for ophthalmic surgery: an international survey use of virtual reality simulation training around the world. Sci Rep. 2025;15. 10.1038/S41598-025-16227-7. PMC12373949 40847049

[6] Nayer ZH, Murdock B, Dharia IP, Belyea DA. Predictive and construct validity of virtual reality cataract surgery simulators. J Cataract Refract Surg. 2020;46:907–12. 10.1097/J.JCRS.0000000000000137. 32541408

[7] Cissé C, Angioi K, Luc A, et al. EYESI surgical simulator: validity evidence of the vitreoretinal modules. Acta Ophthalmol. 2019;97:e277–82. 10.1111/AOS.13910. 30168257

[8] Ferris JD, Donachie PH, Johnston RL, et al. Royal College of Ophthalmologists’ National Ophthalmology Database study of cataract surgery: report 6. The impact of EyeSi virtual reality training on complications rates of cataract surgery performed by first and second year trainees. Br J Ophthalmol. 2020;104:324–9. 10.1136/BJOPHTHALMOL-2018-313817. 31142463

[9] Hu KS, Pettey J, SooHoo JR. The Role of Technology in Ophthalmic Surgical Education During COVID-19. Curr Surg Rep. 2022;10:239. 10.1007/S40137-022-00334-9. 36404795 PMC9662128

[10] Ducloyer JB, Poinas A, Duchesne L, et al. Learning curves of novice residents on cataract surgery simulator: the E3CAPS pedagogic study. BMC Med Educ. 2024;24. 10.1186/S12909-024-06064-Z. PMC11443795 39350156

[11] Thomsen ASS, Bach-Holm D, Kjærbo H, et al. Operating Room Performance Improves after Proficiency-Based Virtual Reality Cataract Surgery Training. Ophthalmology. 2017;124:524–31. 10.1016/J.OPHTHA.2016.11.015. 28017423

[12] Eckardt C, Paulo EB. HEADS-UP SURGERY FOR VITREORETINAL PROCEDURES: An Experimental and Clinical Study. Retina. 2016;36:137–47. 10.1097/IAE.0000000000000689. 26200516

[13] Zhao XY, Zhao Q, Li NN, et al. Comparison of three-dimensional heads-up system versus traditional microscopic system in medical education for vitreoretinal surgeries: a prospective study. BMC Med Educ. 2024;24:290. 10.1186/S12909-024-05233-4. 38491487 PMC10943918

[14] Zhao Xyu, Zhao Q, Li Nning, et al. Surgery-related characteristics, efficacy, safety and surgical team satisfaction of three-dimensional heads-up system versus traditional microscopic equipment for various vitreoretinal diseases. Graefes Arch Clin Exp Ophthalmol. 2023;261:669–79. 10.1007/S00417-022-05850-Z. 36210375 PMC9988774

[15] Srinivasan S, Tripathi AB, Suryakumar R. Evolution of operating microscopes and development of 3D visualization systems for intraocular surgery. J Cataract Refract Surg. 2023;49:988–95. 10.1097/J.JCRS.0000000000001216. 37144641

[16] Ripa M, Kopsacheilis N, Kanellopoulou K, et al. Three-Dimensional Heads-Up vs. Standard Operating Microscope for Cataract Surgery: A Systematic Review and Meta-Analysis. Diagnostics (Basel Switzerland). 2022;12. 10.3390/DIAGNOSTICS12092100. PMC9497825 36140501

[17] Razavi P, Cakir B, Baldwin G, et al. Heads-Up Three-Dimensional Viewing Systems in Vitreoretinal Surgery: An Updated Perspective. Clin Ophthalmol. 2023;17:2539. 10.2147/OPTH.S424229. 37662647 PMC10473403

[18] Sorkin N, Levinger E, Achiron A, et al. Use of a three-dimensional head-mounted digital visualization platform in cataract surgery. Eye (Lond). 2023;37:2905–8. 10.1038/S41433-023-02427-W. 36737520 PMC10517046

[19] Palácios RM, de Carvalho ACM, Maia M, et al. An experimental and clinical study on the initial experiences of Brazilian vitreoretinal surgeons with heads-up surgery. Graefes Arch Clin Exp Ophthalmol. 2019;257:473–83. 10.1007/S00417-019-04246-W. 30645695

[20] Palácios RM, Maia A, Farah ME, Maia M. Learning curve of three-dimensional heads-up vitreoretinal surgery for treating macular holes: a prospective study. Int Ophthalmol. 2019;39:2353–9. 10.1007/S10792-019-01075-Y. 30673952

[21] Helayel H, Bin, Al-Mazidi S, Alakeely A. Can the Three-Dimensional Heads-Up Display Improve Ergonomics, Surgical Performance, and Ophthalmology Training Compared to Conventional Microscopy? Clin Ophthalmol. 2021;15:679–86. 10.2147/OPTH.S290396. 33633441 PMC7901555

[22] Wang K, Song F, Zhang L, et al. Three-Dimensional Heads-up Cataract Surgery Using Femtosecond Laser: Efficiency, Efficacy, Safety, and Medical Education-A Randomized Clinical Trial. Transl Vis Sci Technol. 2021;10. 10.1167/TVST.10.9.4. PMC8340661 34342608

[23] Eckardt C, Ahdab K, Eckert T, Williams GA. Use of Mobile Phones during Heads-up Surgery-A New Way of Teaching Cataract and Vitreoretinal Surgery. Retina. 2019;39(Suppl 1):S191–3. 10.1097/IAE.0000000000002493. 31021901

[24] Iglicki M, Zur D, Khoury M, et al. Technological Influence on Retina Fellows’ Learning Curves: A Prospective Study of Digital versus Analog Microscopes (The BRAVE Study). Ophthalmologica. 2025;248:1–9. 10.1159/000548785. 41056213

[25] Ta Kim D, Chow D. The effect of latency on surgical performance and usability in a three-dimensional heads-up display visualization system for vitreoretinal surgery. Graefes Arch Clin Exp Ophthalmol. 2022;260:471–6. 10.1007/S00417-021-05388-6. 34477929

[26] Dormegny L, Lansingh VC, Lejay A, et al. Virtual reality simulation and real-life training programs for cataract surgery: a scoping review of the literature. BMC Med Educ. 2024;24. 10.1186/S12909-024-06245-W. PMC11529314 39482665

[27] Momin SNA, Memon AS, Malik MB, Mahar PS. Surgical training in ophthalmology: Role of EyeSi in the era of simulation-based learning. J Pak Med Assoc. 2022;72(Suppl 1):S127–9. 10.47391/JPMA.AKU-26. 35202385

[28] Jaud C, Salleron J, Cisse C, et al. EyeSi Surgical Simulator: validation of a proficiency-based test for assessment of vitreoretinal surgical skills. Acta Ophthalmol. 2021;99:390–6. 10.1111/AOS.14628. 33009719

[29] Carr L, McKechnie T, Hatamnejad A, et al. Effectiveness of the Eyesi Surgical Simulator for ophthalmology trainees: systematic review and meta-analysis. Can J Ophthalmol. 2024;59:172–80. 10.1016/J.JCJO.2023.03.014. 37088102

[30] Freeman WR, Chen KC, Ho J, et al. Resolution, Depth of Field, and Physician Satisfaction During Digitally Assisted Vitreoretinal Surgery. Retina. 2019;39:1768. 10.1097/IAE.0000000000002236. 29965938 PMC6310113

[31] Mosca L, Scartozzi L, De Filippis A, et al. 3D Heads-up digital filters for cataract surgery and corneal transplantation. Eur J Ophthalmol. 2024;34:1600–9. 10.1177/11206721241253633. 38710197

[32] Wang Y, Hu Y, Wang D, et al. Overcoming Corneal Opacity Challenges: Visualization Assessment of 3D System With Coaxial Illumination in Cataract Surgery. Am J Ophthalmol. 2025;280:71–8. 10.1016/j.ajo.2025.08.009. 40796024

