# Supplementary material for: Teaching ophthalmic microsurgical skills: conventional microscopy versus heads-up visualization
Source: BMC Med Educ. 2026 Aug 31;26:1403. doi: 10.1186/s12909-026-10269-9 (PMC13528084; doi:10.1186/s12909-026-10269-9)
Supplement: Supplementary file 1 [file 12909_2026_10269_MOESM1_ESM.pdf]

Questionnaire:

Training Effects in the EyeSi® Simulator:

Heads-Up Display vs. Traditional Ocular

Participant ID: ______________________ Date: ______________________

Instructions:
Please rate the following statements separately for the microscope with ocular and the heads-up display.
Scale:
1 = strongly disagree | 2 = disagree | 3 = neutral | 4 = agree | 5 = strongly agree

HEADSUP- 3D:

1. Safety and Understanding
1. I felt in control at all times during the task.
2. I was able to follow the individual steps of the procedure without difficulty.
3. I had sufficient visual feedback to detect errors in time.
4. I felt confident in handling the instrument.
5. I felt able to correct the situation in case of an error.

2. Comfort and Ergonomics
6. My posture during the task was comfortable.
7. I did not experience any physical discomfort (e.g., neck strain or eye fatigue).
8. The visual display was clear and not tiring.
9. I had a good overview of the surgical field.
10. I found it feasible to work with this system for extended periods.

3. Handling and Learning Progress
11. I found the handling of the instruments easy.
12. I was able to control my movements precisely.
13. I noticed rapid progress while performing the task.
14. I was able to identify sources of error during the exercises and learn from them.
15. I felt that this system can help improve my skills in the long term.

4. Teaching and Communication
16. I was able to understand the instructions well during the task.

17. I felt that the instructor was actively involved in the learning process.

OCULAR / MICROSCOPE:

1. Safety and Understanding
1. I felt in control at all times during the task.
2. I was able to follow the individual steps of the procedure without difficulty.
3. I had sufficient visual feedback to detect errors in time.
4. I felt confident in handling the instrument.
5. I felt able to correct the situation in case of an error.

2. Comfort and Ergonomics
6. My posture during the task was comfortable.
7. I did not experience any physical discomfort (e.g., neck strain or eye fatigue).
8. The visual display was clear and not tiring.
9. I had a good overview of the surgical field.
10. I found it feasible to work with this system for extended periods.

3. Handling and Learning Progress
11. I found the handling of the instruments easy.
12. I was able to control my movements precisely.
13. I noticed rapid progress while performing the task.
14. I was able to identify sources of error during the exercises and learn from them.
15. I felt that this system can help improve my skills in the long term.

4. Teaching and Communication
16. I was able to understand the instructions well during the task.
17. I felt that the instructor was actively involved in the learning process.

Comparative Assessment

V1. With which system was it easier overall to complete the tasks?
☐ Microscope with ocular
☐ Heads-up display
☐ No difference

V2. In which system did you feel more confident?
☐ Microscope with ocular
☐ Heads-up display
☐ No difference

V3. Which system did you find more comfortable to use?

☐ Microscope with ocular
☐ Heads-up display
☐ No difference

V4. In which system did you experience greater learning progress?
☐ Microscope with ocular
☐ Heads-up display
☐ No difference

V5. In which system was interaction with the instructor better?
☐ Microscope with ocular
☐ Heads-up display
☐ No difference

V6. Which system would you recommend for beginners in microsurgery?
☐ Microscope with ocular
☐ Heads-up display
☐ No difference

V7. Were you able to transfer skills learned on the heads-up display to working with the ocular microscope?
☐ Yes
☐ No
☐ Partially

V8. Were you able to transfer skills learned on the ocular microscope to working with the heads-up display?
☐ Yes
☐ No
☐ Partially

Open Feedback

Do you consider the use of a surgical simulator to be a useful preparation for real surgical procedures?

____

What did you particularly like about the microscope with ocular?

____

What did you particularly like about the heads-up display?

____

What difficulties did you encounter with the respective systems?

____

What suggestions for improvement would you make for future use?

____
